# An Ethnographic Study of the Social Context of Migrant Health in the United States

**DOI:** 10.1371/journal.pmed.0030448

**Published:** 2006-10-24

**Authors:** Seth M Holmes

**Affiliations:** 1 Department of Anthropology, History, and Social Medicine, School of Medicine, University of California San Francisco, San Francisco, California, United States of America; 2 Department of Anthropology, University of California, Berkeley, California, United States of America; University of Birmingham, United Kingdom

## Abstract

**Background:**

Migrant workers in the United States have extremely poor health. This paper aims to identify ways in which the social context of migrant farm workers affects their health and health care.

**Methods and Findings:**

This qualitative study employs participant observation and interviews on farms and in clinics throughout 15 months of migration with a group of indigenous Triqui Mexicans in the western US and Mexico. Study participants include more than 130 farm workers and 30 clinicians. Data are analyzed utilizing grounded theory, accompanied by theories of structural violence, symbolic violence, and the clinical gaze. The study reveals that farm working and housing conditions are organized according to ethnicity and citizenship. This hierarchy determines health disparities, with undocumented indigenous Mexicans having the worst health. Yet, each group is understood to deserve its place in the hierarchy, migrant farm workers often being blamed for their own sicknesses.

**Conclusions:**

Structural racism and anti-immigrant practices determine the poor working conditions, living conditions, and health of migrant workers. Subtle racism serves to reduce awareness of this social context for all involved, including clinicians. The paper concludes with strategies toward improving migrant health in four areas: health disparities research, clinical interactions with migrant laborers, medical education, and policy making.

## Abstract 2

A qualitative study of migrant Triqui Mexicans in the western US and Mexico shows that structural racism and anti-immigrant practices lead to poor working and living conditions, and poor health.

## Editors' Summary

**Background.:**

For centuries, recent immigrants have experienced poorer living and working conditions than more established inhabitants, which in turn means that the health of immigrants is often worse. Immigrants often take on the very lowest-paid jobs. One might suppose that in more recent years the increasing prosperity of countries such as the United States and those of western Europe would have reversed this trend. But as recently as 2005 the New York–based Human Rights Watch published a report entitled “Blood, Sweat and Fear,” which documented appalling conditions for the mostly immigrant workers in the US meat and poultry industry. In the UK also, legislation has recently been introduced to try to regulate the activity of “gang masters” who control large groups of immigrant workers. This legislation was triggered by public horror about the deaths in 2004 of 21 immigrant cockle pickers who drowned in Morecambe Bay in Lancashire. A group of workers at particular risk of poor conditions because of the seasonal and uncertain patterns of work are those who work as farm laborers.

**Why Was This Study Done?:**

There are relatively few studies that have looked in detail at the pattern of health problems among migrant farm workers in the US. Understanding the working conditions of these workers would be of help in understanding more about their health problems and, in particular, how to prevent them. One problem is that few of these workers are seen in the usual health-care settings; few of them have health insurance.

**What Did the Researchers Do and Find?:**

The paper's author spent 15 months with a group of indigenous Triqui Mexicans as they migrated around the western US and Mexico working on farms. He used a type of research called qualitative research, which involved observing and interviewing more than 130 farm workers and 30 health workers on farms and in clinics. He found that working and housing conditions were organized according to ethnicity and citizenship, and that there was an unofficial hierarchy, with undocumented indigenous Mexicans having the worst health. Even worse, migrant farm workers were often blamed for their sicknesses by those in charge of them or those from whom they sought help.

**What Do These Findings Mean?:**

The author concludes that “structural racism and anti-immigrant practices determine the poor working conditions, living conditions, and health of migrant workers.” Furthermore, it seems that “subtle” racism among all involved, including clinicians, reduces awareness and perhaps even allows tacit acceptance of these patterns of health. It seems that targets for specific health interventions for these workers will need to be closely integrated with a broader approach to improving migrant health including medical education and policymaking.

**Additional Information.:**

Please access these Web sites via the online version of this summary at http://dx.doi.org/10.1371/journal.pmed.0030448.

- Migration Dialogue regularly consolidates news related to immigration around the world
- Global Exchange has information related to fair trade, CAFTA, and other related current events
- United Farm Workers has information related to working conditions of migrant laborers
- PCUN has information related to migrant laborers in the Pacific Northwest
- The Border Action Network has information related to the US-Mexico border
- Border Links provides education and experiential learning related to the US-Mexico Border
- Tierra Nueva and the Peoples Seminary provide social services for migrant laborers in the Pacific Northwest and education related to the lives of migrant workers
- The Pesticide Action Network of North America provides information related to pesticides and health
- The Pesticide Education Center provides detailed lists of the contents of pesticides and their health effects
- The Center for Comparative Immigration Studies conducts research and education projects related to international migration
- Human Rights Watch publishes and campaigns on many issues, including conditions for workers, such as that on the US meat-packing industry
- European Research Centre on Migration and Ethnic Relations has a range of information concerning migrants

## Introduction

Labor migration is a significant phenomenon throughout the world, with high economic, political, medical, and human stakes. The UN Population Division estimates that there are 175 million migrants in the world, 46% more than a decade ago [1]. Worldwide, the majority of migrant laborers are of a minority ethnicity in the country in which they work, most live in poverty and suffer poor health, and significant numbers are undocumented.

What is often framed as “the migrant problem” [2,3] in the US has received great political, journalistic, and medical attention in the past few years. Recent research estimates that there are 293 million residents in the US, 36 million of whom are foreign-born and 10.3 million of whom are unauthorized [4,5]. According to the 2000 US Census, there were 9.2 million Mexican-born US residents, including 2.3 million naturalized US citizens, 2.1 authorized immigrants, and 4.8 undocumented immigrants [6]. It is estimated that there are 1 million indigenous Mexicans from the state of Oaxaca in the US, mostly Mixtec, Zapotec, and Triqui people [7]. Approximately 95% of the agricultural workers in the US were born in Mexico [8] and 52% are undocumented [9]. The average age of agricultural workers is 29 years, with very few older than 60 years [10,11] and the vast majority of these individuals and families live below the poverty line [9,11,12].

Most researchers agree that inequalities in the global market make up the primary driving force of labor migration patterns (e.g., [12]). Mexico's average minimum wage is US$4.12 per day and varies by region, with the lowest minimum wage in southern Mexico, from which come most indigenous Mexican migrants to the US. In contrast, the US federal minimum wage is $5.15 per hour, while it is $6.75 in California and $7.15 in Washington state. Regardless of the lack of parity in economic power between Mexico and the US, the North American Free Trade Agreement (NAFTA) deregulated all agricultural trade in 2003 except for corn and dairy products, which will be unprotected in 2008 [13]. The Mexican government complains that since NAFTA's initial implementation in 1994, the US has raised farm subsidies by 300% [13]. Throughout the 1990s, Mexico, on the other hand, has reduced financial supports for corn producers, millions of whom are indigenous peasants for whom corn cultivation is the primary source of income [13]. Various Mexican organizations are pressing the Mexican government to renegotiate NAFTA so that more farm owners and workers will not be forced by poverty to emigrate for wage labor [13]. In various rural parts of Mexico, rebel groups have risen up, some armed and some not, to demand a change to the economic marginalization and geographic displacement justified by the rhetoric of “development” and “free trade” (e.g., [14,15]).

Mexican laborers, often called “illegal aliens” in the US, are often victims of negative prejudice and violence, including murders by civilian vigilante squads, so-called “beaner raids” by off-duty Marines, BB gun shootings by white American youth, deportations of sick workers by company owners under the guise of taking them to the hospital, the paying of entire farm labor crews in wine and illicit drugs, and pesticide poisoning by company crop dusters (for specific examples, see [2,4,16–19]). They are blamed for everything from unemployment rates to state budget deficits [20] in efforts to pass bills (such as California's “Save Our State” initiative in the 1990s, a similar 2005 initiative in Arizona, and various US Congress bills in 2006) that bar undocumented immigrants from public services, including health care. In such political debates about immigrants, it is rarely acknowledged that these laborers are actively recruited by US employers to take jobs that US citizens most often are unwilling to fill, and that the laborers pay sales taxes as well as the federal, state, and local taxes taken out of their paychecks [5,21–24].

In 1994, the US Border Patrol launched Operation Gatekeeper to deter migrants from crossing the southern border by utilizing more agents, more barriers, and more technology. According to critics, this has simply moved the location of crossings to more deadly areas that are less visible to border area residents [25]. Already by August, 2005 had become the deadliest year on record, with 385 recorded border-crossing deaths, surpassing the previous record of 383 set in 2000 [25]. In addition, undocumented immigrants report that *coyote* (unauthorized border-crossing guide from Mexico to the US) fees have risen to approximately US$2,000. Nonetheless, the “new nativism” [26,27] active in the US calls for further militarization of the border. During the summer of 2005, more than 1,000 private volunteer militiamen, calling themselves “Minutemen,” began patrolling 23 miles of the Arizona border [28]. California governor Arnold Schwarzenegger commended the Minutemen for doing a “terrific job” [25], while others consider it dangerous and illegal vigilantism [29].

Previous medical and public health research shows that migrant farm workers have significantly worse health statistics than other populations. Such statistics are somewhat unreliable, due to the difficulty of studying a largely invisible population. Estimates of the migrant farm laborer population in the US range from 750,000 to 12 million, though most approach 10 million [11]. In addition, most morbidity and mortality data are skewed lower due to undocumented workers' fear of reporting health problems, poor enforcement of labor and health policies in agriculture, as well as the fact that many Latin American migrant laborers return to their home countries as they age or become disabled, which leads to a “healthy worker bias” [30]. Regardless of these issues, previous research shows that health disparities related to migrant farm workers fall into the areas of ethnicity, citizenship, and social class. According to recent research, Latino children have twice the death and hospitalization rates from pedestrian injury than do white children in the US, and Latino adults have lower rates of preventive medicine screening [8]. A recent Institute of Medicine report indicates that all ethnic minorities receive definitively lower-quality health care in the US [31].

In addition, several studies show that the health status of immigrants declines with increasing time in the US. Such health markers as obesity, serum cholesterol, tobacco smoking, alcohol use, illicit drug use, mental illness, suicide, and death by homicide increase between first- and second-generation Mexican immigrants in the US [8,30]. The nutritional value of immigrants' diets also decreases significantly during the first year in the US [30]. Undocumented status further increases allostatic load due to, among other things, crossing the “most violent border in the world between two countries not at war with one another” as well as ongoing fear of immigration and other authorities [7].

Beyond ethnicity and immigration status, the class position of Mexican migrant farm workers is also associated with their decreased health status. Agricultural work has a high fatality rate, with 21.3 deaths per 100,000 workers per year, compared with the overall worker rate of 3.9 [10]. In addition, agricultural workers have increased rates of nonfatal injuries, chronic pain, heart disease, many cancers, and chronic symptoms associated with pesticide exposure [10]. There is also an increased risk of stillbirth and of congenital birth defects in children born near farms [10,32]. To further specify class position, migrant and seasonal farm workers suffer the poorest health status within the agriculture industry. Migrant and seasonal workers have increased rates of many chronic conditions, such as HIV infection, malnutrition, anemia, hypertension, diabetes, chronic dermatitis, fatigue, headaches, sleep disturbances, anxiety, memory problems, sterility, blood disorders, dental problems, and abnormalities in liver and kidney function [11]. This population has an increased incidence of acute sicknesses such as urinary tract and kidney infections, lung infections, heat stroke, anthrax, ascariasis, encephalitis, leptospirosis, rabies, salmonellosis, tetanus, and coccidioidomycosis [32,33]. Tuberculosis prevalence is six times more common in this population than in the general US population [30]. Finally, children of migrant farm workers show high rates of malnutrition, vision problems, dental problems, anemia, and excess blood lead levels [32].

Despite their worse health status and a correlated need for more health and social services, migrant farm workers face many obstacles to access such services. Farm workers are entirely or partially excluded from worker's compensation benefits in all but 15 states [33]. The Fair Labor Standards Act of 1938 guaranteed minimum wage, time-and-a-half wage for overtime, and restricted child labor, but this did not apply to farm workers. Amendments in 1966 ostensibly extended eligibility to farm workers, but disqualified the majority by excluding such categories of workers as those on small farms and those paid piece wages. The majority of farm workers are also excluded by the Social Security Act and its later amendments from benefits related to unemployment. In addition, even though migrant housing conditions are addressed in Housing Act of 1949 and Occupational Safety and Health Act of 1970, living conditions in labor camps continue to be appalling. Finally, farm workers were denied the right to collective bargaining under Wagner Act of 1935, which has changed in only a very few states. Furthermore, even existing provisions for farm workers are regularly violated.

Only 5% of migrant farm workers have health insurance, contrasted with 84% of US residents overall [30,34]. Migrant laborers are less likely than other groups to obtain preventive care, with 27% never receiving a routine physical exam, 25% never having a dental check-up, and 43% never receiving an eye exam [11]. Although there is a federal Migrant Health Program funding migrant clinics, it is estimated that this program serves only 13% of the intended population [11]. Finally, undocumented status and the inter-state migratory nature of their lives means that less than one-third of migrant women qualify for Medicaid, despite living well below the poverty line [31]. Many migrant workers in the US go through many hardships to return to Mexico for health care [31], and they cite economic, cultural, and linguistic reasons for this choice.

The social science research cited above indicates that Mexican migrant workers in the US are the focus of many forms of prejudice and violence. The health research brings to light significant health disparities related to undocumented Mexican migrant workers, specifically along the lines of ethnicity, citizenship, and social class. However, it is not yet understood how prejudice—specifically, institutional racism and anti-immigrant prejudice—might influence such health disparities. There has been very little research related to the ethnic make-up of migrant workers in general, especially with relation to indigenous Mexican migrants. This study aims to fill these gaps by identifying ways in which the social context of indigenous, undocumented migrant farm workers' affects their health status, well-being, and medical care.

This research investigates the social forces in the web of causation of ill health among migrant workers in the US by reporting in-depth qualitative research data and analysis from an extended ethnographic case study with undocumented, indigenous Mexican migrant laborers in the western US and Mexico. In addition, the investigator analyzed ways in which the social origins of sickness are obscured by an individualizing medical gaze as well as societal normalizing notions of essential ethnic difference.

## Methods

In order to address these research questions, this study employs the classic anthropological technique of participant observation [35], supplemented by tape-recorded, semi-structured, in-depth interviews in farms as well as in clinics and hospitals frequented by migrant farm workers. Because the study question relates to subtle forms of prejudice, assumption, and meaning that are often difficult to assess with quantitative methods or interviews alone, this study makes use of the above standard qualitative research methods utilized over the long term. Anthropological methods, such as those described below in the case of migrant health, are critical to investigating social disparities in health in vivo without simplifying the complex reality in which they are embedded. Specifically, participant observation involves long-term immersion in a particular social and cultural context. The researcher participates in everyday life during an extended period of time, while observing interactions and listening to conversations in order to identify significant practices, political economic forces, and cultural concepts. The investigator regularly records events and conversations in detailed field notes. While this methodology may include data from interviews and surveys, it differs significantly from many other methods of research in that it is performed and analyzed within the situational knowledge provided by long-term participation, observation, and relationship.

Tape-recorded interviews were conducted with five to ten members of each of the groups of primary study participants outlined in Table 1, except for vigilante members and Border Patrol officers (who refused to be recorded but agreed to be interviewed and for notes to be taken). The researcher is fluent in English and Spanish and speaks and understands limited Triqui Alto. Interviews were conducted by the investigator in English or Spanish when either of these was fluently spoken and understood by the participant. Given the extreme lack of Triqui translators, the investigator utilized the translation help of other primary study participants in order to interview the few participants who spoke only Triqui Alto.

**Table 1:**
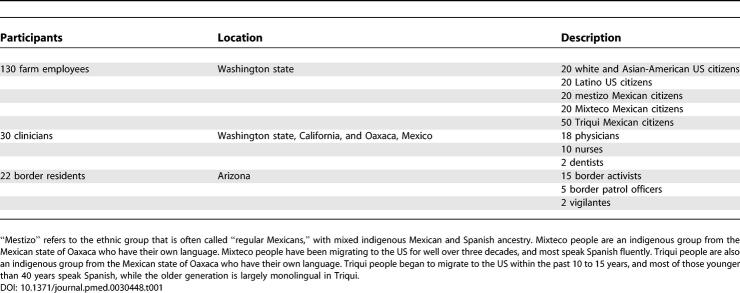
Primary Study Participants

The participant sample was selected in order to balance the need for the organic development of relationships within participant observation and the desire for a representative sample. The selection of participants on the Tanaka Farm will serve as an example. First, this farm was selected for the study because it contains populations that represent the ethnic and citizenship make-up of much of the agricultural workforce in the US [36–38], thus increasing the generalizability of the findings. Second, the investigator was able to build rapport with the farm management due to his prior acquaintance with several area residents.. The sample of participants was selected to represent each of the primary labor, ethnicity, and citizenship positions on the farm. Within each of these positions, the individuals whom the investigator was able to observe in multiple meetings were consented and included as study participants. The general principles of choosing participants from each of the primary ethnicity and citizenship categories whom the investigator was then able to observe in multiple temporal and social contexts were repeated throughout the multi-sited field research.

**Figure 1:**
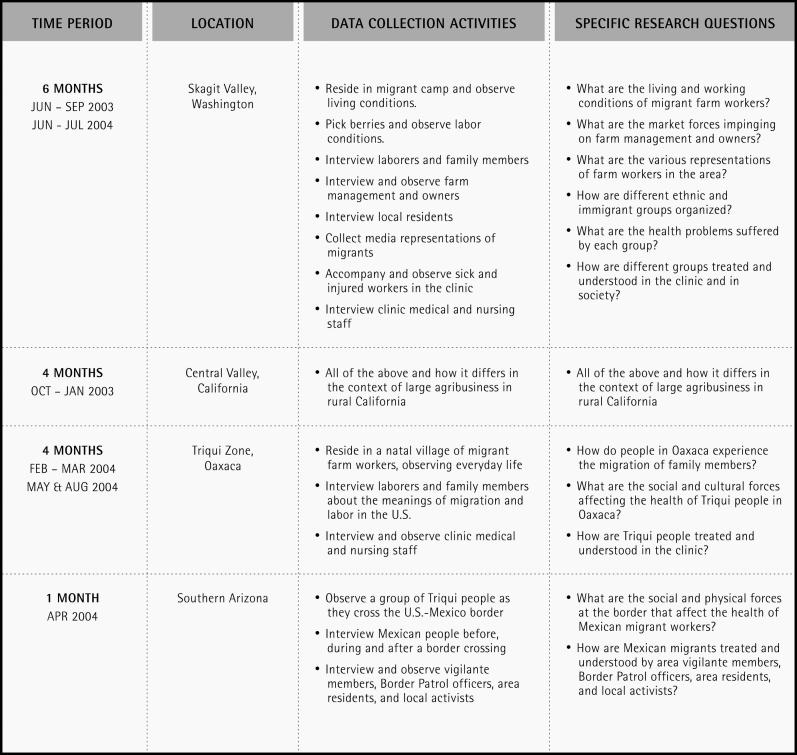
Summary of Field Work

**Figure 2:**
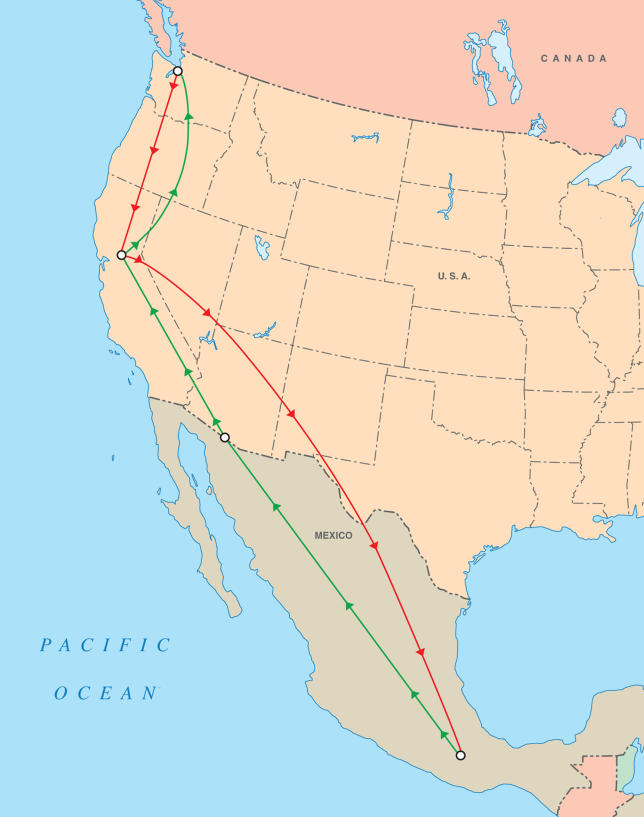
Map of Migration Route This map shows the route of migration field research followed by the anthropologist, from the Skagit Valley of Washington state to the central Valley of California, the mountains of Oaxaca, Mexico, the Arizona borderlands, and then back to central California and Washington state. (Illustration: Natalie Davis)

The research was conducted along the lines of “follow the people” multi-sited fieldwork [39] full-time for 15 months throughout a migration circuit with a group of indigenous Triqui Mexicans starting in an agricultural community in Washington state, moving to central California, next moving to their hometown in the mountains of Oaxaca, migrating across the border into Arizona, and finally returning to Washington state (see Figure 1). Six months were spent living in a migrant camp, picking berries, as well as observing and interviewing in migrant clinics and hospitals in Washington state. Four months were spent living with Triqui migrant workers in a slum apartment, pruning vineyards, as well as observing and interviewing in migrant clinics and hospitals in central California. Four months were spent living with a family, planting and harvesting corn, and observing and interviewing staff in the local medical clinic in a village in the mountains of Oaxaca. One month was spent hiking through the desert, meeting with and interviewing Border Patrol agents, local residents, activists, and vigilante members in Arizona (Figures 1 and 2).

More than 3,000 pages of field notes from observations and experiences, oral histories and transcribed interviews, as well as photographs, surveys, clinical medical charts, newspaper and other media clips comprise the data analyzed in this study. In this project, data were analyzed according to the primary foci of living and working conditions, ethnic relations, immigration status, health status, and medical care, as well as prejudices and stereotypes.

A general overview of the initial stages of data analysis is provided by the model of grounded theory [40], which can be particularly useful in participant-observation studies. The most intensive phase in each research site can be understood as the scientific method of hypothesis testing via observation done in an iterative process over the longue durée. Frequently during fieldwork, the primary investigator systematically analyzed and coded field notes and interviews in order to test the primary hypotheses of the study and develop more precise questions for the next rounds of interviews. This method allows for ongoing contextual development of more and more precise hypothesis testing. Fieldwork notes and transcribed interviews were coded utilizing Atlas.ti software. The analysis process includes coding of data through cycles of increasing precision. The data with a single code were compiled and analyzed for their characteristics and meanings. Then, data were coded axially, focusing on connections among categories. Data analysis also entailed a research verification technique called triangulation. This involves collecting several kinds of data from the same sources over time as well as from independent sources in order to verify the validity of research findings and to diminish distortion due to self-report alone.

This analysis was performed with cross-checking by 15 scholars in the fields of cultural anthropology, medical anthropology, and medical sociology. During the analysis and writing phases of the study, meetings were scheduled with seven senior scholars individually and as a group with the eight researchers in the Violence in the Americas Writing Group (see Acknowledgements section) to discuss developing analyses of field notes and interview transcriptions that had been sent to them earlier. Consensus regarding analysis was achieved through discussion of the analyses—including themes—arrived at separately by each of the individuals mentioned above. In addition, several study participants were invited to discuss the conclusions of this project. The invitation of critiques and analyses by study participants is an increasingly common practice in cultural and medical anthropology that works to increase the validity of findings by minimizing the a priori bias of the outsider. The investigator, however, holds the final responsibility for the conclusions presented in this article.

The theoretical framework of this study falls within the subfield of critically interpretive medical anthropology [41,42]. This subfield of medical anthropology seeks to remain close to the origins of cultural and medical anthropology in the US by focusing on the interpretation of local meanings and experiences while linking these interpretations with an analysis of larger social, political, and economic forces. This framework is especially helpful in answering research questions that attempt to understand the inter-relationships between the micro illness experiences of individuals and the macro social and cultural forces influencing those experiences. The specific concepts within this field that will be employed in this paper will be described in the Results and Discussion sections. This study was approved by the Committee on Human Research at the University of California San Francisco. The identity and research aims of the investigator were made clear throughout the fieldwork. Names and identities of study participants have been changed without altering the nature of the data. Consent for photographs was obtained from all participants in the manner suggested by the Committee on Human Research at the University of California San Francisco.

## Results

### Ethnicity and Citizenship Hierarchies in Farm Labor

The Tanaka Farm, in which several months of fieldwork were performed in Washington state, serves as an extended case study in order to understand the effects of prejudice on the health of farm workers. The first, general phase of fieldwork yielded the contextual data [43] that will be presented in this section.

**Figure 3:**
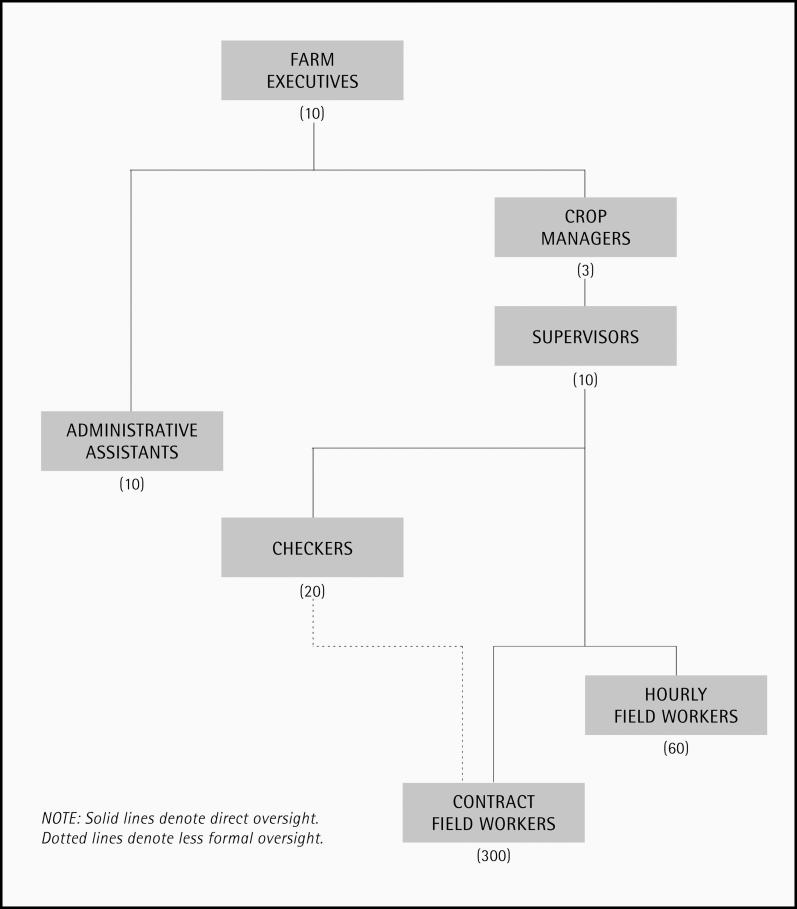
Labor Hierarchy on the Tanaka Farm

The Tanaka Farm is a relatively small family farm, with executives focused on growing the business for future generations and keeping agricultural land in Washington state. This farm employs more than 400 workers at the peak of picking season and approximately 50 during the rest of the year. On a practical and explicit level, employees on the farm plant, harvest, and process berries, supporting the published goals of the company. On another level, the structure of farm work inheres a hierarchy reflecting the inequalities in US society at large—specifically, those organized around ethnicity and citizenship. Duties, privileges, as well as exposures to weather, pesticides, and other dangers differ from the top to the bottom of this hierarchy (see Figure 3).

**Figure 4:**
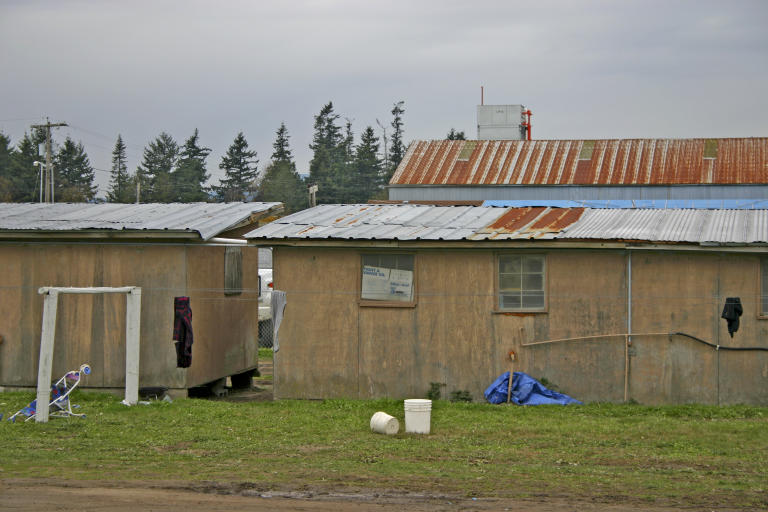
Labor Camp on the Tanaka Farm Each of the Tanaka Farm's labor camp units, called “cabins” by farm executives, is split in half, with each side housing one family.

The broad contours of the structure of labor on the Tanaka Farm follow. The top executives work seated behind desks in private offices and live in their own houses, some with panoramic views. All are white or Asian-American. The administrative assistants who work seated at desks in public spaces as well as the teenagers who stand outside checking weights and time cards live in relatively simple houses near the farm. They are almost entirely white, with a few US Latinos. The other workers live in one of three labor camps. Each labor camp is made up of shacks, the average of which is 10 feet by 15 feet with one or two mattresses, one small refrigerator, two camping-style gas stoves, one table with a bench, and a small sink with one hose each of hot and cold water. The first camp holds almost 50 people and is located 100 feet from the road. Each shack has heating, insulation, and wooden roofs under the tin metal sheets. Here live the field bosses who walk outside supervising the pickers, almost entirely Latino US citizens, along with one Mixteco from the state of Oaxaca in Mexico. The second camp holds approximately 100 people and is located a few hundred feet from the road. Most units here have a wooden roof under the tin metal sheets, though none have heating or insulation. Here live primarily apple and raspberry pickers, as well as several strawberry pickers. The residents of this camp are made up almost entirely of undocumented mestizo Mexicans, along with several Mixtecos and a few Triquis, also from Oaxaca. The third camp, located several miles from the farm headquarters down a back road, holds 250 people. The shacks here have tin roofs without wood, heating, or insulation. Here live the majority of the strawberry pickers, primarily Triqui indigenous Mexicans, as well as several Mixtecos and two indigenous people from the Mexican state of Chiapas (Figure 4).

The ethnic and citizenship hierarchy seen here—white and Asian-American US citizen, Latino US citizen or resident, undocumented mestizo Mexican, and undocumented indigenous Mexican—is common in North American farming [36–38,44]. The relative status of Triqui people below Mixtecos can be understood as a difference in perceived indigeneity. Many farm workers and managers indicated in interviews that they believed that the Triqui were more “purely indigenous,” “more simple,” and “less civilized” than other groups.

**Figure 5:**
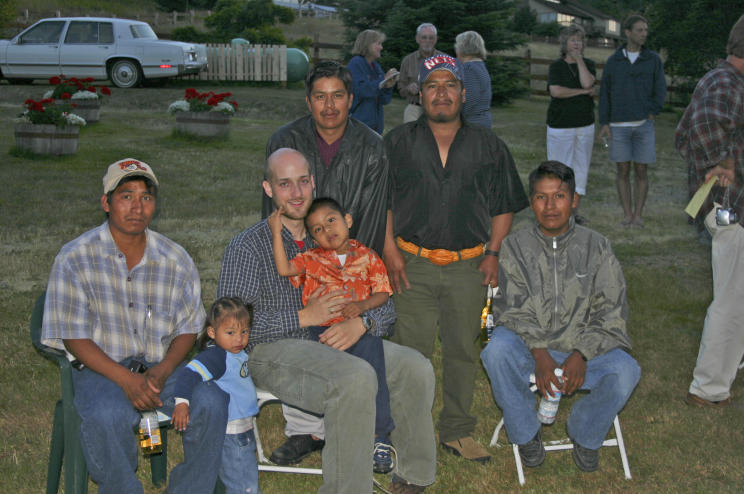
The Anthropologist with Triqui Migrant Workers in Washington State The anthropologist with Triqui migrant workers in Washington state.

In many ways—ethnicity, citizenship, social class—the investigator did not take the appropriate position in the labor hierarchy. In order to answer the research questions, the anthropologist placed himself in the housing and occupations of the Triqui undocumented immigrants. This, then, added experiential data beyond the observations and interviews. The farm executives treated the investigator as someone out of place, giving him special permission to keep his job and shack even though he was never able to pick the minimum weight. They joked and talked with him as he picked, treating him like a respected form of entertainment. On the other hand, the Oaxacan berry pickers treated him with a mixture of respect and suspicion. Many wondered why there was a *gabacho chakuh* (bald, white American) picking berries. Many suggested that the investigator might be a spy for the police, the border patrol, or the US government. Others stated that he might be a drug smuggler looking for a good cover (Figure 5).

After sharing a meal in the labor camp, a Triqui man named Samuel made a statement representative of many interactions between the berry pickers and the investigator. He mused, “Right now you and I are the same; we are poor. But, later you will be rich and live in a luxury house *(casa de lujo)*.” The anthropologist explained that he did not want a luxury house, but rather a small, simple house. Samuel clarified, “But you will have a bathroom on the inside, right?”

### Health Disparities and Health Care

The first question in the hypothesis-testing phase of research follows: How does the above ethnic and citizenship hierarchy in agricultural labor relate to health status and health care? With time and observation, it became clear that the complex of ethnicity, citizenship, labor, and housing maps onto a hierarchy of health status and suffering. The further down the ladder from Euro-American to indigenous Mexican one is positioned, the less control over time one has, the more degrading treatment by supervisors one receives, the more physically taxing one's work is, and the more exposed one's body is to weather and pesticides. As enumerated in the Introduction section, disparities in many areas of health in the US fall along this hierarchy of ethnicity, labor, and citizenship.

The Triqui people inhabit the bottom rung of the pecking order on the Tanaka Farm. The relationship between their position in the farm labor structure and their health constitutes a representative case in point. As described above, the Triqui berry pickers live in the coldest, wettest shacks. They hold the most stressful, humiliating, as well as physically strenuous and dangerous jobs picking strawberries. Occupying the bottom of the labor hierarchy, Triqui strawberry pickers bear an unequal share of health problems, commonly experiencing back and knee degeneration, diabetes, dental problems, and often giving birth prematurely to low–birth weight infants (see, e.g., [7,9,10,11,30,32,33]). Four common health problems among Triqui pickers that will be explored further below include occupational injury and pain, somatization, substance abuse, and trauma. Triqui experiences of health care will also be examined.

**Figure 6:**
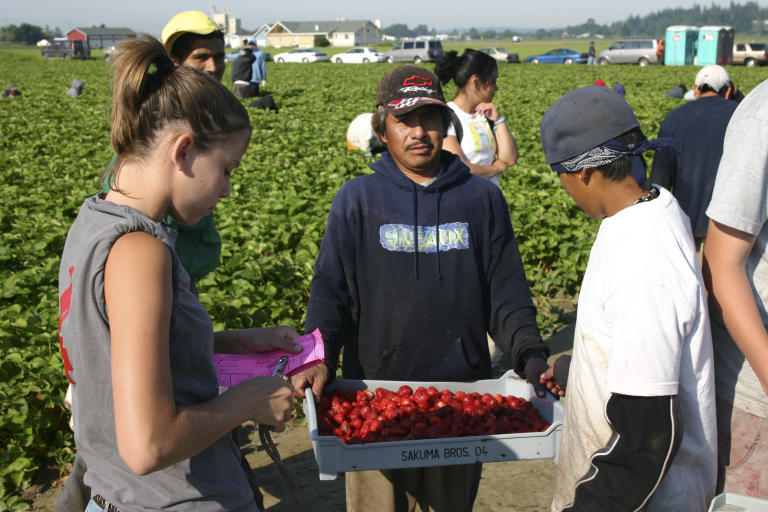
Teenage Checker Punches the Weight of Berries Brought in by a Picker A teenage checker punches the weight of berries brought in by a picker.

**Figure 7:**
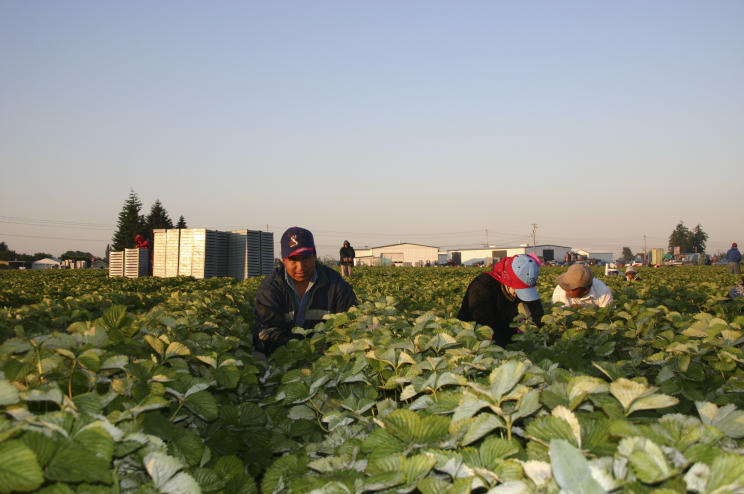
Triqui People Bent Over Picking Strawberries in Washington State Triqui laborers picking strawberries on the Tanaka Farm in Washington state.

Strawberry pickers must bring in 50 pounds of de-leafed berries every hour. Otherwise, they will be fired and kicked out of the camp. In order to meet this minimum weight requirement, they take few or no breaks from 5:00 a.m. until the afternoon or evening when that particular field is completed. Often, they are reprimanded nonetheless and called *perros* (dogs), *burros* (burros), *Oaxacos* (a derogatory term for “Oaxacan”), or *indios estupidos* (stupid Indians). Many do not eat or drink anything before work so that they do not have to take time to use the outhouse. They work as hard and fast as they can, picking and running with their buckets of berries to the white teen checkers. Meanwhile, the white teenagers stand to the side, talking and laughing, sometimes throwing berries at each other in jest, and occasionally hurling berries at Triqui pickers with statements made at high volume such as, “Eat it!,” or simply, “No!”(Figure 6). One of the first Triqui pickers the investigator came to know, named Abelino, explained the experience of picking in the following way (Figure 7):

“… You pick with both hands, bent over, kneeling like this [demonstrating with both knees fully bent and his head bowed forward]. Your back hurts; you get knee pains and pain here [touching his hip]. Well, when it rains, you get pretty mad and—and—you have to keep picking. They don't give lunch breaks. You have to work every day like that … You suffer a lot in work.”

During the fieldwork, the anthropologist picked once or twice a week, providing valuable experiential data for analysis [45]. After each day of picking, the investigator experienced gastritis, headaches, as well as knee, back, and hip pain for two to three days afterward. Triqui strawberry pickers, on the other hand, worked seven days a week, rain or shine, until the last strawberry was processed. In order to more fully explore the effects of social context, including prejudice, on the health and health care of Triqui people, three individual cases will be highlighted.

### Abelino: Work Injury and Chronic Pain

Due to the long hours and difficult conditions of strawberry picking, many workers complained of back, hip, and knee pain. In order to further understand the experiences and meanings of such common pain, one extended case study will be presented here. Abelino, a Triqui father of four who was mentioned above and lived near the anthropologist in the labor camp, came to the US across the deadly desert border to work long enough to save approximately US$10,000 in order to return to his hometown in the mountains of Oaxaca and build a concrete house for himself and his extended family. He summarized the need to migrate one day, stating:

“In Oaxaca, there's no work for us. There's no work. There's nothing. When there is no money, you don't know what to do. And shoes; you can't get any. A shoe like this [pointing to his tennis shoes] cost about 300 Mexican pesos. Per day, they're paying 30 or 40 pesos. You have to work two weeks to buy a pair of shoes … We have to migrate to survive. And we have to cross the border, suffering and—and—and walk two days and two nights, sometimes five days to get here and work and support the American people. Because they don't work like we do. They just get rich working a job—a light job—like the shops, the offices, but they don't work in the field. But we Mexicans from many Mexican states come here to maintain our families. We want to get permission to enter just for a harvest season and then return to our country … And we come here and it is a little better, but you still suffer in the work … Coming here with the family and moving around to different places, we suffer. And the children miss their classes … and don't learn well. Because of this, we want to stay here only for a season with [legal documents] and let the children study in Mexico. Do we have to migrate to survive? Yes, we do.”

One Saturday, Abelino experienced acute, sharp pain in his knee when he turned while picking strawberries in the row next to where his wife and oldest daughter were also picking. After continuing his work in vain hopes that the pain would go away, he told his field boss about the incident. The boss simply said, “OK,” and drove away without any follow-up. Unsure of what to do, Abelino attempted to keep picking.

Two days later, work was abruptly canceled without explanation from the supervisors, and Abelino went to a local urgent care clinic. During the course of the next year, he ended up seeing four doctors and a physical therapist, usually without a translator. During this time, he limped around camp, taking care of his kids while his wife and oldest daughter continued picking in the fields.

The urgent-care doctor he first saw explained that Abelino should not work, but should rest and let his knee recover. The occupational health doctor he saw the following week said Abelino could work but without bending, walking, or prolonged standing. Abelino went to the farm office to ask for lighter work of this sort. The bilingual receptionist told him in Spanish in a frustrated tone, “No, because no,” and did not let him talk with anyone else. After a few weeks, the occupational health doctor passed Abelino to a reluctant physiatrist who told Abelino that he must work hard picking strawberries in order to make his knee better. She told Abelino that he had been picking incorrectly and hurt his knee because he “didn't know how to bend over correctly.” Once Abelino had recovered, this doctor explained to the researcher that Abelino no longer felt pain, not because he got better, but because the picking season was over and he could no longer apply for worker's compensation. Two years later, Abelino still tells the anthropologist that he has occasional knee pain and that “the doctors don't know anything *(no saben nada)*.” Knee and back pain continue to be the most common health complaints among pickers on the Tanaka Farm (see also [10]).

### Crescencio: Somatization and Substance Use

Crescencio, another Triqui father who lived in the same camp, approached the investigator later in the summer and asked for medicine for headaches. He explained that every time a crew boss called him names on the job, made fun of him, or reprimanded him unfairly he got an excruciating headache in the center of his head. He told the researcher that the headaches made him more prone to anger with his wife and his children and that he wanted treatment so that he would not be at risk of abusing them. He had seen a few doctors in Mexico and the US as well as a traditional Triqui healer, all to no avail. The only remedy he found to make the headache go away was drinking 24 beers. He resorted to this form of self-medication a few times in an average week. A week later, he saw one of the doctors in the local migrant clinic to seek help, but left disappointed. In an interview, the physician explained her perspective:

“Well, yes, he thinks that he is the victim and thinks that the alcohol or the headache makes him beat his wife … but really he is the perpetrator and everyone else is the victim. And until he owns his problem, he can't really change. I'm on the CPS [Child Protective Services] subcommittee and so I know a lot about domestic violence, and what we've seen is that nothing really works, none of these migraine medicines or anything, but to put people in jail because then they see a show of force. That's the only thing that works because then they have to own the problem as theirs and they start to change. It's a classic case of domestic abuse. He came to see me once, and I told him to come back two weeks later after not drinking. But he didn't come back two weeks later; instead, he came back a month later and saw not one of our best doctors but an OK doctor, one of our locums. Apparently, he told the doc something about when people at work tell him what to do, it makes him mad and that's what gives him a headache. Obviously he has issues. He needs to learn how to deal with authority. We referred him to therapy. Do you know if he's going to therapy?”

While the specific details of Crescencio's story are unique, his problem is representative of the common phenomena of somatization and substance use among migrant laborers. When asked to enumerate the most common problems of this population, several physicians and nurses in the local migrant clinic in Washington state responded that the issue they saw most commonly was depression in the form of somatization and/or substance abuse. Commonly, when somatic complaints of unclear etiology and substance use were explored further during interviews of pickers in the context of rapport and trust, it became clear that many of the important proximal determinants of such suffering involved social and cultural factors. The most common of such factors included disrespect from supervisors and area residents, lack of choices for work, lack of opportunities for social advancement, fear of being deported, and grieving the distance from family members and home in Oaxaca state.

### Bernardo: Trauma and Political Violence

**Figure 8:**
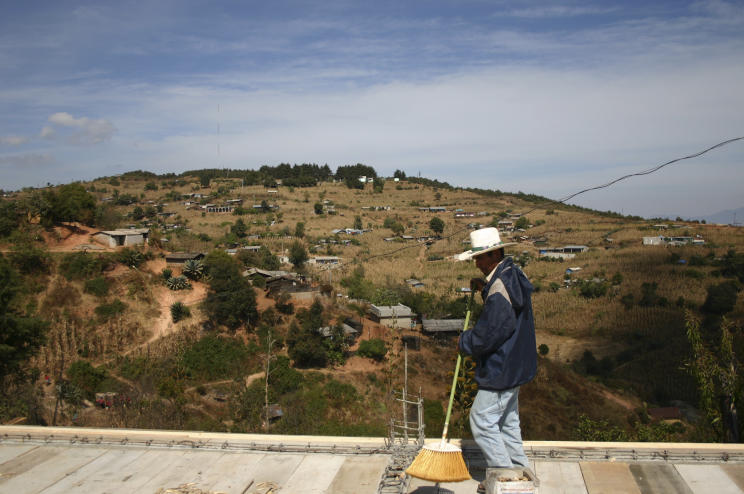
A Hometown of the Triqui People in Oaxaca State, Mexico The hometown in the mountains of Oaxaca, Mexico, of many Triqui migrant laborers.

Violence and trauma make up another important health-related factor experienced by every Triqui picker in one way or another, from commonly reported border violence, to frequent violence at the hands of the Mexican military in Oaxaca state, to regularly experienced violence in the workplace. Some of the myriad health effects of such experiences of violence are made clear in the extended case study of Bernardo. Bernardo, a Triqui man who indicates that he is now somewhere between 62 and 80 years old, was one of the first Triqui people to come to the US in the 1980s. He stopped working on the farm and moved back to Oaxaca when he became a US resident in the 1986 amnesty. He has spent five months each summer since then working in a fish processing plant in Alaska in order to support himself, his wife, and his sister. The rest of the year, he returns to the mountains of Oaxaca, to be with his family. This area, sometimes called “the Triqui Zone” of Oaxaca, is reputed to be violent. There have been several small land wars between Triqui villages and neighboring mestizo towns. There have also been conflagrations between a local movement cum political party, known as the Unified Movement for Triqui Progress (MULT), and the political party that has been in power in Oaxaca for dozens of years, the Institutional Revolutionary Party (PRI) (Figure 8).

During part of the field research in Oaxaca, the investigator lived and worked with Bernardo and his family in the small city of Juxtlahuaca. This family is originally from a small Triqui town further into the mountains; however, the family's land was on the edge of town next to a different village, with whom there was a slow, ongoing land and political war. Bernardo described this situation:

“There have been many deaths. Oh! Many deaths! … Maybe eight, maybe ten in the last two months ... They kill between political parties. There is a lot of danger here. If you say something and don't realize someone heard you and they are hidden, all of a sudden, ‘Pow!' or a knife and you are dead. I can't go out at night, even if we need something. Not at night, no! A lot of danger. There is a lot of danger here. During the day is fine. I go to the market and to the doctor, but not at night. I have fear. A lot of danger, yes, yes.”

Bernardo's family, along with many others, moved to Juxtlahuaca to escape the violence. With the money Bernardo made migrating to the US, the family was able to build a house and start a very small store in Juxtlahuaca.

One night, Bernardo asked the investigator if he knew of any good medicine for Bernardo's stomach. Bernardo explained that he had experienced stomach pain for approximately eight years. He stated, “My stomach does not like food any more. I don't have the desire *(ganas)* to eat. It hurts to eat.” Before he goes to Alaska each spring, his doctor in Juxtlahuaca gives him a long series of vitamin shots and “shots to give hunger” so that he has enough energy to work. When he returns from Alaska, he is weak and thin and is given another long series of the same shots to recover from the work. The following description of his pain was punctuated by groans and accented by many hand gestures:

“It gives me such a pain! Right here [pointing to his stomach], such a pain, and it goes up … It jumps and jumps like chords jumping, like this, like this [rapidly opening and closing his hands] … I wake up and my stomach hurts; ay! It was hard like this bench is hard … So, I mash my stomach with a soda bottle. I mash, mash, mash, mash here, mash here. And it helps a little. But, ay! I can't stand it. I can't eat! Nothing! Each time I eat it hurts; but it hurts. But I hold out [me aguanto], I hold out, and I hold out until work is over. It feels like it is twisting, twisting like so [rotating his hands quickly].”

Bernardo explained that he has lost weight over the past several years and feels weak each morning when he goes to work his family's cornfields at 5:00 a.m. He has to force himself to eat a tortilla and an egg before working his fields.

In response to the question, “Why does your stomach hurt?”, Bernardo explains that it is because he has worked so hard all of his life. Bernardo has lived the migrant life since the age of eight, working from dawn until dusk seven days a week in northern Mexico or the US, then returning to work hard on his family's land in Oaxaca state. “So much working *(tanto trabajar)* wears out a body,” he explained with a weak smile.

Yet, when asked more specifically why the pain started eight years ago, Bernardo added:

“Also … the soldiers punched and kicked me many, many times. Punched like this [making a fist and punching into the air], here in my stomach. Ah! But many beatings [chingadazos] … Until there was blood all over. Because of the movement [the MULT]. People said rumors against us and the soldiers, the blue ones, came and beat me up.”

Eight years ago, Bernardo was kidnapped and tortured by the Mexican federal police in charge of narcotics enforcement (“the blue ones”), who are supported by US Drug Enforcement Agency money. Bernardo was beaten several times and put in prison. There, he was allowed no medical help and resorted to drinking his own urine as a remedy to help his abdomen heal. Furthermore, he reported that he was denied food many of the days while in captivity. Members of the “blue military,” as he called them because of their uniform color, told him that he had been kidnapped under the suspicion that he was part of the MULT, even though the movement has no history with drugs. After several months, the mayor of Juxtlahuaca wrote, signed, and stamped an official paper stating that Bernardo had done no wrong, and he was finally released from prison.

In an interview with the researcher, Bernardo's physician in Oaxaca state told the investigator that Bernardo has a peptic acid problem like gastritis. He suggested that this gastrointestinal problem was due to eating “too much hot chili, too much fat, and many condiments.” He continued, “They [indigenous people] also don't eat at the right time, but wait a long time in between meals.” The physician gives Bernardo an H2-blocker to decrease his peptic acid levels. He stated that proton-pump inhibitors would work better, but they are too expensive for Bernardo to afford. He also recommends that Bernardo eat milk and yogurt. Finally, the physician gives injections of vitamin B12 in order to treat what he diagnosed as Bernardo's neuropathy. He explained that this neuropathy was due to the fact that indigenous people “bend over too much at work and bend too much in their sleep.”

While most Triqui people have not been wrongfully imprisoned and beaten by the federal police, all of them are affected in one way or another by political violence. Beyond crossing the politically violent border between the US and Mexico [46], Triqui migrant laborers deal with land wars and political violence in their hometowns every time they return home. For some, like Bernardo, this leads to somatic complaints, for some it leads to poor mental health, and for still others it leads to mortal injury (e.g., five Triqui people involved in this research project were shot in Oaxaca, four of them fatally, during the field research; see also [47,48]). Much of the violence in southern Mexico is directed against indigenous people, especially against those involved in movements working toward equality. This violence affects indigenous people not only in Mexico, but also when they are in the US as migrants.

### Racism, Naturalization, and Internalization

The second primary question of the hypothesis-testing phase follows: How has the order of ethnic, citizenship, labor, and health inequalities become seen as so normal that it is rarely questioned or challenged? Though there were many different prejudices, stereotypes, and metaphors employed by interviewees to make sense of these inequalities, one of the most prevalent involved perceptions of natural differences among the bodies of different ethnicities. When asked why very few Triqui people were harvesting apples, the field job known to pay the most, the Tanaka Farm's apple crop supervisor explained in detail that “they are too short to reach the apples, and, besides, they don't like ladders anyway.” He continued that Triqui people are perfect for picking berries because they are “lower to the ground.” When asked why Triqui people have only berry-picking jobs, a mestiza Mexican social worker in Washington state explained that “a los Oaxaqueños les gusta trabajar agachado [Oaxacans like to work bent over],” whereas, she told me, “Mexicanos [mestizo Mexicans] get too many pains if they work in the fields.” In these examples and the many other responses they represent, perceived bodily difference along ethnic lines serves to justify or naturalize inequalities, making them appear purely or primarily natural and not also social in origin. Thus, each kind of ethnic body is understood to deserve its relative social position.

**Figure 9:**
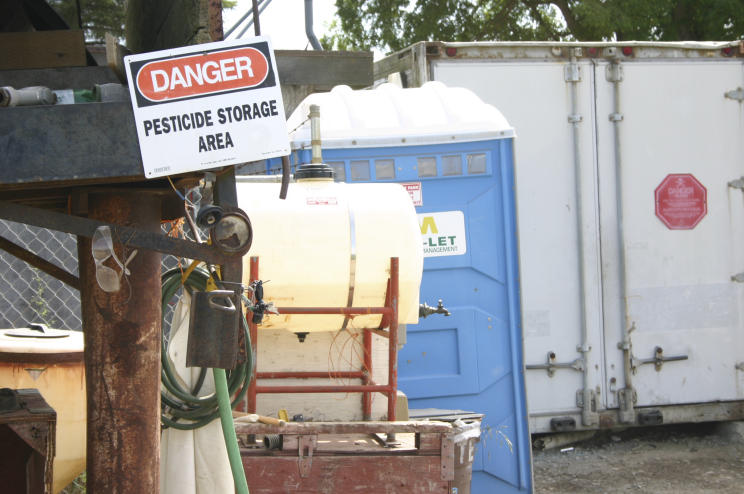
Pesticide Containers Surround Hand-Washing Station and Outhouse Containers of pesticides surround one of the hand-washing stations and outhouses on the farm.

At the same time that area residents and other farm employees naturalized the position of Triqui pickers, it was also rare for the Triqui pickers to question the hierarchies described above. On one of the days when the investigator picked strawberries, a tractor with long metal extensions spraying something in the air drove through the same field that was being picked. The anthropologist asked a supervisor what it was. “Do you really want to know? You sure you want the truth? Dangerous insecticides,” he said, shaking his head. In addition, one of the primary hand-washing and outhouse stations on the edge of the field was located within an area of several large canisters marked with pesticide danger signs. Strawberry pickers in Washington state worked every day without gloves as the visible pesticide residues dissolved in the mixture of strawberry juice and morning dew that would stain their hands dark maroon for days. If they ate anything, they ate it in the fields, while picking, without washing their hands to save time and make the minimum weight. The only education for pickers about pesticides came from a short warning cassette tape in monotone Spanish played inaudibly in one corner of a huge warehouse full of 100 or more workers and their children during the picker orientation (Figure 9).

The same week as the spraying described above, the researcher, along with several Triqui pickers, watched a video about the health-related dangers of pesticides. Afterward, one commented matter-of-factly that “pesticides affect only white Americans *(gabachos)* because your bodies are delicate and weak,” whereas “we, the Triquis, are strong and hold out *(aguantamos)*.” The others agreed. Here, Triqui people internalize their position in the labor and health hierarchy through their pride in perceived bodily differences. Triqui people naturalize the labor hierarchy utilizing similar perceptions of ethnic difference. Because of these perceptions, the migrant body is seen as belonging in its position in the very system that then leads to its deterioration.

**Figure 10:**
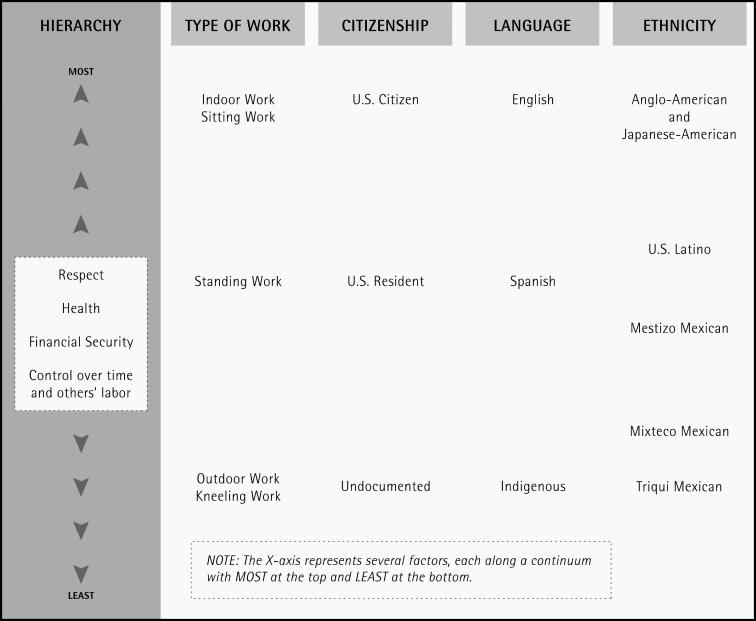
Conceptual Diagram of Hierarchies on the Farm

Figure 10 summarizes, utilizing a conceptual diagram, many of the themes resulting from this field research. The *y*-axis represents respect, health, financial security, and control over one's own time as well as control over others' labor. The various columns along the *x*-axis show differences among types of work, citizenship statuses, languages, and ethnic groups. Gender is another important variable that is not considered here due to space constraints, but should be examined further in future research.

## Discussion

In conclusion, this research reveals insights into the relationships between ethnic and citizenship prejudices and the health status and health-care experiences of migrant laborers. The health and well-being of such workers are influenced on several levels from international to domestic, as well as local to occupational and clinical. To summarize the results of this study, working and housing conditions are organized from best to worst among farm employees according to ethnicity and citizenship status: from white or Asian-American US citizen to Latino US citizen or resident to undocumented mestizo Mexican to undocumented indigenous Mexican. This ethnicity–immigration–labor hierarchy determines a correlated hierarchy of health status, with the undocumented Triqui Mexicans having the worst health. Yet, those involved—including medical professionals—are largely unaware of the social context of health. Subtle forms of racism, specifically understandings of ethnic bodily differences, function to justify and naturalize the place of each group in the labor and health hierarchies (Holmes, In Press).

The choice to use qualitative methods presents advantages as well as disadvantages. The ethnographic study design does not allow for a determination of the strength of association among different factors studied. Similarly, ethnographic methods do not allow for the calculation of prevalence and incidence of various forms of suffering. The relatively small study population and its geographical specificities limit the ability to generalize to other populations of migrant workers worldwide. The recruitment of the majority of the study participants from the Tanaka Farm labor pool inevitably excluded those workers who had become sick or injured enough to be unable to return to their work, and this may, therefore, lead to an underestimation of suffering.

As shown by various researchers in the field of social studies of science and technology (e.g., [49–51]), the social position and social interactions of scientific investigators influence their own research as well as how their results are viewed by others. This is true not only in laboratory science, but also in social science. In the case of ethnographic research such as the present study, the interactions of the investigator and the participants become data for analysis. Much of this kind of data is considered above. In addition, the researcher affected the populations studied in several other practical ways. For example, his presence during the housing search of the Triqui people in central California appeared to make landlords of substandard housing nervous and, thus, more likely to reject these Triqui people as tenants. His presence in Washington state played a significant role in raising awareness of the desires of Triqui laborers to have English as a Second Language (ESL) courses as well as gravel covering the alternately dusty and muddy labor camp driveways. Through the coalition of various community members and organizations, ESL courses were offered in the labor camps during the second summer of the research and the primary camp driveways were graveled. The investigator's presence in Oaxaca meant that the families with whom he lived could communicate—via sending and receiving small packages of food and clothing—more easily with their loved ones across the border, though it also meant that the children in these families were sometimes made fun of by their peers. As a final example, the investigator was involved in raising awareness of the desire of several of the Triqui high school students to have a Triqui Alto dictionary in order to prevent the continued loss of their language. Through various circumstances, a linguistics doctoral student from one of the investigator's home institutions is currently working on this project (C. Dicanio, personal communication).

Ethnographic research did, however, allow for in-depth investigation into the dynamics of complex social forces—such as meanings of ethnicity and experiences of inequalities—that are not amenable to epidemiologic or survey studies alone. Participant observation allowed the investigator to move beyond worker, employer, or medical professional report in order to observe and experience interactions and conditions firsthand. Ethnography uniquely allowed for the investigation of these multiple forms of data and multiple points of view in order to produce data and analysis that more fully represent the complex reality it studied. Finally, long-term involvement in the lives of marginalized groups that may often be suspicious of outsiders allowed for rapport building that increases the validity of data.

### Structural Features of Inequalities

As the qualitative data above suggest, these hierarchies are not conscious or willed on the part of the farm owners or managers. Much the opposite, larger structural forces as well as the anxieties they produce drive these inequalities. The Tanaka Farm executives are ethical people who have a vision of a good society that includes family farming. Perhaps instead of blaming the growers, it is more appropriate to understand them as human beings trying to lead ethical, comfortable lives, committed to the family farm in the midst of an unequal, harsh system. The corporatization of US agriculture and the deregulation of international free markets squeeze growers such that they cannot imagine increasing the pay of the pickers or improving the labor camps without bankrupting the farm. In this case, structural violence is enacted by market rule and then channeled through international and domestic racism, classism, sexism, and anti-“illegal” immigrant sentiments. Wacquant [52] points out the analytical pitfalls of overly generalized, nonspecific use of the term “structural violence.” This term is employed here to mean simply the violence—visible as injury to body and self-respect—enacted by social structures, primarily exploitative economic relations. Engels [53] explains that the effects of unequal social structures can be “as violent as if [the economically exploited] had been stabbed or shot.”

Abelino, Crescencio, Bernardo, and other farm workers endure forms of suffering that are directly and indirectly influenced by social and political forces. The late modern system of free-trade capitalism has compounded global inequities, leading southern Mexico into a deepening economic depression. This poverty is one of the primary factors producing the local land wars as well as the survival-seeking out-migration of able-bodied workers. Once in the US, these people are relegated to some of the most unhealthy labor positions. The political alliances of the Mexican military, with its ties to the US federal government via financing, have translated into a repression of the many movements seeking redistribution of power in a more equitable fashion. The torturing of members of indigenous rights movements by the military functions not only to deepen the suffering of its victims, like Bernardo, but also to reinforce the neoliberal economic system and thus deepen global economic inequalities.

### Symbolic Features of Inequalities in Society and the Clinic

In order to further understand the naturalization of the inequalities described above, Pierre Bourdieu's theory of symbolic violence proves effective [54–56]. According to Bourdieu, symbolic violence is the naturalization and internalization of social asymmetries. He explains that humans perceive the social world through lenses issued forth from that very social world. Thus, we misrecognize the social order as natural. The structures of inequalities comprising the social world are thus made invisible and taken for granted for all involved. The concept of symbolic violence also inheres a sense of internalization such that one does not perceive only others, but also oneself, as belonging in particular social and economic locations. As seen in the qualitative data above, perceived bodily differences along ethnic lines comprise one of the lenses through which symbolic violence is enacted such that each ethnic group is understood to deserve its relative social position. The structural violence inherent to segregated labor on the farm is so effectively erased precisely because its disappearance takes place at the level of the body, and is thus understood to be natural. This was seen in the data retrieved from area residents, farm employees, and medical professionals.

Physicians and nurses in migrant clinics work hard under relatively poor conditions without access to state-of-the-art medicines and instruments and are often frustrated by the obstacles in a system with irregular funding and virtually no insurance coverage. One physician in Washington state explained one of many obstacles in her work: “Most [migrants] don't have any insurance, so that's even harder 'cause you start them on a medication and you know they are just going to be off it again wherever they go next.” Despite the hard work and dedication of clinicians in the field of migrant health, the Triqui people regularly stated that the “doctors don't know anything *(no saben nada)*.” What explains this apparent discrepancy?

In *The Birth of the Clinic,* Michel Foucault describes what he calls the “clinical gaze” [57]. Foucault explains that there was a change in clinical medicine with the advent of cadaveric dissection in the early modern era. Whereas physicians used to focus on the words of the patient, the symptoms as expressed by the patient, they began to focus on the isolated, diseased organs, treating the patient more and more as an object, a body. As would be expected within this paradigm, the medical professionals described above saw the Triqui bodies in their offices, yet were unable to engage the human and social context leading to their suffering. These clinicians, like most medical professionals, were not trained to see the social determinants of health problems. Thus, it was unavoidable that they would fall into the trap of utilizing a narrow lens that decontextualizes sickness. Thus, many of the most proximal determinants of suffering were left unacknowledged, unaddressed, and untreated.

Beyond this acontextual gaze, physicians in North America today are also taught to see behavioral factors in health—such as lifestyle, diet, habits, and addictions. Behavioral health education has been added as part of the laudable move to broaden medical education within the paradigm of biopsychosocial health first described by George Engel in 1977 [58]. However, without being trained to consider the global political economic structures and local prejudices that shape the suffering of their patients, health professionals are equipped with only biological and behavioral lenses to understand suffering.

As seen in the cases above, well-meaning clinicians often blame the sickness on the patient—e.g., the assumed incorrect bend while picking, the supposed trouble with authority, or the “incorrect” eating and sleeping habits—without appreciating the local hierarchies and international forces that place their patients in injurious working conditions in the first place. Ironically, the progressive move to include behavioral health in medical education without the correlate inclusion of social context may be precisely that which leads clinicians to blame the victims of social suffering. In addition, stereotypes of Mexican migrant workers—e.g., that the men are alcoholics and abuse their wives—are supported by lenses that decontextualize the suffering and marginalization often at the root of their poor health behaviors. Even those health professionals acutely aware of the social determinants of health may resort to biological and behavioral explanations as a defense mechanism against what they experience as overwhelmingly hopeless. The relationship between undocumented Mexicans and the migrant clinic is further convoluted by the clinic's own affiliation with the US government via funding and regulations. This affiliation foments intermittent rumors and fear among Triqui workers that clinic staff may turn them in to the Border Patrol.

### Implications for Medicine and Beyond

Drawing on the ethnographic data above, this article will close with recommendations toward improving the health of migrant farm workers in four areas: research into ethnic and immigration status disparities in health, clinical interactions with individual migrant laborers, medical education, and policy making.

First, in order to further understand ethnic and citizenship disparities in health, researchers must take into account the international context of migration. Research remaining limited to local and domestic factors will inevitably fall short of describing the reality for immigrants. Qualitative and quantitative researchers must find ways to explore the implications of racism and anti-immigrant prejudices in the development and maintenance of health disparities. Further dissecting the mechanisms by which social inequalities become taken for granted is an especially critical area for such research, especially in the current political climate. Only in this way will people become able to see the social determinants of such inequalities and capable of imagining and working toward alternatives. In-depth, ethnographic methods appear to be especially capable of describing the webs of causation of health disparities without losing the complexity of the context in which they are embedded.

Second, the ethnographic data indicate several steps clinicians can take in order to provide more appropriate and competent care to this population (see also [59]). Given the difficulties of clinical encounters in a medical system that is practiced—as it is in the US—on an individual level, often semi-controlled by funders, and usually in unrealistically short time allotments, clinicians must be creative within the constraints of their context of practice, while also considering means to change these constraints. The first step for clinicians to provide more appropriate care is screening, identifying an individual patient as a migrant laborer. The clinician may ask such questions as: Where is the patient's hometown? How long have they been in the area and in their present post of employment? What are their work and housing arrangements? Does the patient feel that she has the ability to negotiate with her employer and landlord over these conditions? For undocumented immigrants, a physician's acknowledgement of these circumstances and validation of their right to safe conditions may be a powerful intervention in and of itself (see also [59]). In addition, workers at risk of depression and substance use may be identified and referred for further help. The second step is to consider the contributing etiologies of a patient's sickness from not only biological and behavioral but also social domains [60–62]. The clinician can ask herself: How do international and local inequalities, occupational structures, economic forces, racial inequalities, and other social and cultural factors influence the health and sickness of the patient? This identification and acknowledgement of social determinants of disease not only allows for a more precise understanding of a patient's sickness, but also prevents unfairly blaming the patient for their sickness. This, then, avoids inflicting further psychological harm at the same time that it allows for the building of a more effective therapeutic relationship. Finally, in their mandate to alleviate suffering, physicians are called to attend to all of the determinants of a given patient's sickness. Instead of addressing solely the biological and behavioral etiologies by offering only medical, surgical, and psychological therapy, this mandate indicates that physicians should also tackle social determinants. This could take the form of advocating for the amelioration of specific workplace hazards, educating migrant laborers about their rights, and pointing them in the direction of available services in the area, or becoming involved in policy making (as described below).

Third, in order for the clinical changes above to take effect, medical education must be broadened to reflect the multifaceted etiology of disease. Those readers who work in medical curricula will recognize that the vast majority of training programs spend most of their time on biological or pathophysiological etiologies and a small part of their time on psychological or behavioral components. Very few programs spend any appreciable time teaching social analysis to medical trainees. This study shows that the social context is critical to the development of sickness and suffering among migrant workers, and this is likely true among other populations. The lack of training to recognize social determinants of disease relates partially to a paucity of capable educators in this area. Most academic physicians do not have adequate training in social analysis themselves in order to teach it to their students. Most social scientists do not know how to speak the language of medical students and, thus, either come off as ethereal or attempt to simplify their message and inadvertently make it boring. Medical educators must attempt new methods for social medicine education—such as the case-based program of social medicine grand rounds at the University of California San Francisco—and new programs and further funding must be developed for training social medicine educators who are both knowledgeable in social theory and conversant in clinical medicine.

Policy making will be the final area of consideration of implications from this study. In the specific case of Triqui migrant laborers and in the world at large, medicine and sociopolitical inequalities are interrelated. As Rudolf Virchow argued in the 19th century after his investigations in population health, “Medicine is a social science, and politics is nothing else but medicine on a large scale” [63]. He also said that, due to the unequal distribution of morbidity and mortality along socioeconomic classes and the calling of physicians to alleviate suffering first and foremost, “physicians are the natural attorneys of the poor” [ibid]. With these reasons in mind and considering the moral authority of medical personnel in much of the world, it is essential for physicians to consider and become involved in politics. In this effort, it is important to take cues from the long history of health and community activism that has revolved around migrant farm workers' rights as related to the present research findings, from the United Farm Workers nationally to the Tierra Nueva Family Resource Center in western Washington state; from Marion Moses and the Pesticide Education Center's online database to the Food Justice Alliance's roundtable discussions; and from the No More Deaths Movement of Arizona to the programs of California Rural Legal Assistance.

Mexican migration to the US is complexly determined by international market policies, global power inequalities, as well as regional and local prejudices and fears. The nexus of political economic structures driving migration with legal structures barring entry to immigrants and widespread anti-immigrant sentiments proves unhealthy and dangerous. This nexus is becoming especially volatile at the time of this writing [64], with the US Congress discussing bills that would give local police the authority to investigate and enforce federal immigration laws, federal policy makers proposing hundreds of miles of new fence and significantly increased military personnel along the already militarized US-Mexico border, and the George W. Bush administration discussing a poorly defined temporary worker program that appears to make the power differential between worker and employer even greater than it is already. While policy change is critically necessary, any proposal that does not address the primary political and economic determinants of migration is doomed to fail. Amelioration of the social suffering inherent to undocumented labor migration requires a careful consideration and confrontation of historical, political, economic, and symbolic factors producing and reproducing this phenomenon. The further deregulation of international and domestic trade—such as the currently proposed Central American Free Trade Agreement [65]—should be questioned critically and vocally for its potential effects on marginalized people and their ability to survive in their home communities without being forced to migrate for work. Policies supporting the labeling of products according to the labor conditions under which they were made—such as those agricultural products from farms with United Farm Workers contracts, thus allowing consumers to make decisions related to this information—should be considered strongly. The growing Domestic Fair Trade Working Group is working toward such a labeling program in the US [66].

The Triqui people involved in this study indicated repeatedly that they want to keep their homes in Oaxaca and work in the US one season at a time. They support a fair temporary worker system that does not increase the power differential between employers and employees, as many fear the George W. Bush administration's current plan would do. The poorly outlined Bush proposal appears to link legal permission to be in the US with one specific employment contract, thus giving that particular employer the virtual power to deport via firing [67]. This inability to change employers is reminiscent of the recent convictions of slavery related to the horrendous conditions in which many migrants are already forced to work (see also [68]). A fairer program would allow employee mobility when working contracts are undesirable, unfair, or unfulfilled. The US government and US society gain much from migrant laborers and give little back beyond prejudice, criminalization, and suffering [16,22,69]. This dishonest relationship, similar to many labor migration systems around the world, must be acknowledged and changed. Medical professionals have the calling and the authority to work toward this change.

## Supporting Information

Text S1 Translation of the Abstract into Spanish (22 KB DOC)
