# Supplementary material for: An Ethnographic Study of the Social Context of Migrant Health in the United States
Source: PLoS Med. 2006 Oct 24;3(10):e448. doi: 10.1371/journal.pmed.0030448 (PMC1621098; doi:10.1371/journal.pmed.0030448)
Supplement: Supplementary file 1 [file pmed.0030448.sd001.doc]

ABSTRACT

RESUMEN

  Fondo:
  La salud de los trabajadores migrantes en los Estados Unidos es extremadamente mala. Este ensayo intenta identificar las maneras, a través de las cuales, el contexto social de los trabajadores migrantes afecta tanto a su salud como a su tratamiento médico.
  
  Metodolofía y resultados:
  A lo largo de quince meses, se convivió y entrevistaron a migrantes indígenas de la etnia triqui, desde su lugar de origen en el estado mexicano de Oaxaca, hasta en el Oeste de EEUU., donde se entrevistaron más de 130 trabajadores y 30 médicos y enfermeras. Las entrevistas fueron realizadas tanto en los campos de cultivo como en clínicas y la información obtenida fue analizada mediante la "grounded theory" y las teorías de la violencia estructural, simbólica y mirada clínica. Este estudio revela que las condiciones laborales y de vida en los campos dependen de la etnia y la ciudadanía de los trabajadores, jerarquía que determina las desigualdades en la salud, siendo los indígenas mexicanos indocumentados los más afectados.
  
  Conclusiones:
  El racismo estructural y las prácticas anti-inmigrantes determinan las malas condiciones laborales, de vida y por tanto de salud de los trabajadores migrantes. El racismo sútil los minimisa, incluyendo el de los médicos y enfermeras. Este ensayo concluye con cuatro áreas cuya finalidad es mejorar la salud de los migrantes, éstas son: investigación de las desigualdades de salud, las relaciones entre los migrantes y la clínica, la educación médica y los retos políticos.
